# Macromolecular crystallography beamline X25 at the NSLS

**DOI:** 10.1107/S1600577514003415

**Published:** 2014-04-08

**Authors:** Annie Héroux, Marc Allaire, Richard Buono, Matthew L. Cowan, Joseph Dvorak, Leon Flaks, Steven LaMarra, Stuart F. Myers, Allen M. Orville, Howard H. Robinson, Christian G. Roessler, Dieter K. Schneider, Grace Shea-McCarthy, John M. Skinner, Michael Skinner, Alexei S. Soares, Robert M. Sweet, Lonny E. Berman

**Affiliations:** a Photon Sciences Directorate, Brookhaven National Laboratory, PO Box 5000, Upton, NY 11973-5000, USA

**Keywords:** beamline, mini-κ, Pilatus 6M, PXRR, macromolecular crystallography, wBPM

## Abstract

Beamline X25 at the NSLS is one of the five beamlines dedicated to macromolecular crystallography operated by the Brookhaven National Laboratory Macromolecular Crystallography Research Resource group. This mini-gap insertion-device beamline has seen constant upgrades for the last seven years in order to achieve mini-beam capability down to 20 µm × 20 µm. All major components beginning with the radiation source, and continuing along the beamline and its experimental hutch, have changed to produce a state-of-the-art facility for the scientific community.

## Abstract 2

A description of the upgraded beamline X25 at the NSLS, operated by the PXRR and the Photon Sciences Directorate serving the Macromolecular Crystallography community, is presented.

## 1. Introduction   

X25 is a facility beamline operated by the Macromolecular Crystallography Research Resource group (PXRR) and the Photon Sciences Directorate at the 2.8 GeV National Synchrotron Light Source (NSLS) at Brookhaven National Laboratory (BNL). It has been in operation since 1990 and became solely dedicated to macromolecular crystallography in 2004. At that point, the beamline was re-tasked to use only monochromatic radiation which prompted a re-configuration of the optical elements along the beamline to redefine the beam characteristics for its singular purpose. Major upgrades have been on-going in order to promote the use of a mini-beam to match the trend of smaller biological samples. This beamline is used in tandem with X29 (Shi *et al.*, 2006 ▶), also an undulator beamline, and together they accommodate most of the life science users who make up 40% of the users coming to this synchrotron light source.

## 2. Beamline overview   

X25 began operation in 1990 based on a hybrid wiggler radiation source, and was configured flexibly to allow endstation operation using monochromatic beam or white beam, focused or unfocused (Berman *et al.*, 1992 ▶). It served a variety of physical and biological science experimental programs spanning from high-*q* resolution elastic scattering to Laue diffraction. With the re-dedication of the beamline experimental program to focus entirely on monochromatic beam macromolecular crystallography (MX) in 2004, an opportunity arose to rebuild the radiation source, beamline, endstation and controls. The new optimization for this singular purpose supplanted the original generic configuration. Thus there were equipment implementations, over a period of a few years beginning in 2006, when the original hybrid wiggler, the water-cooled double flat crystal monochromator and the original toroidal white-beam double focusing mirror were replaced. Simultaneously, a state-of-the-art micro-diffractometer replaced the original four-circle diffractometer in the experimental station, and eventually a pixel array detector (PAD) replaced the original CCD area detector.

**Figure 1:**
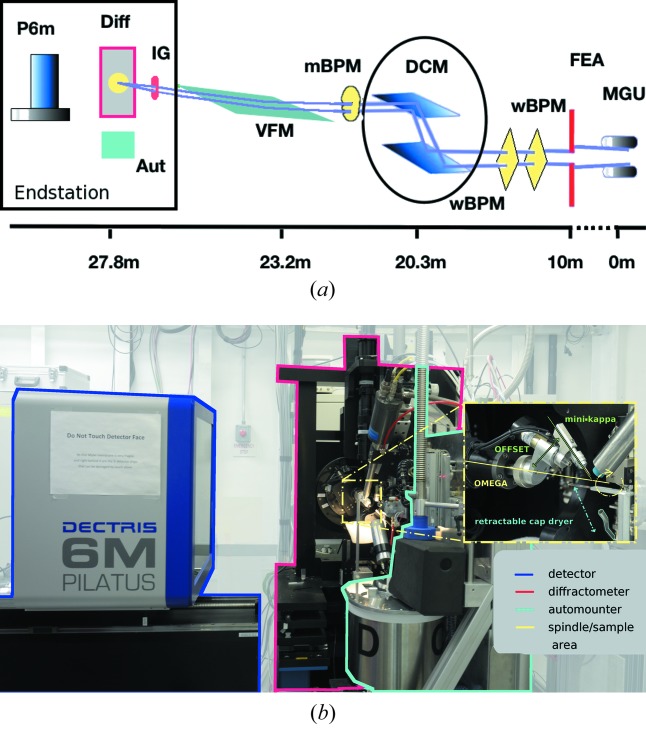
(*a*) The NSLS X25 beamline layout highlighting the locations of MGU: mini-gap undulator; FEA: the water-cooled front-end aperture; wBPM: white-beam position monitor; DCM: sagittally focusing double-crystal monochromator; mBPM: monochromatic-beam position monitor; VFM: vertically focusing mirror; IG: ionization gauge; DIFF: diffractometer assemble (ω spindle, slit apertures, cryostream, fluorescent counter); Aut: automounter; P6M: Dectris pixel-array detector. (*b*) View of the endstation. Close-up of the mini-κ goniometer; at its maximum open angle, κ is offset from ω by 42°.

The new in-vacuum miniature gap undulator consisting of 18 mm periods (55 in total) delivers beam across an energy range of 6.8−13.5 keV allowing typical SAD or MAD experiments from the Fe to the Br edges (Ablett & Berman, 2007 ▶). A water-cooled aperture defines the size of the emitted photon beam. Next, a thin graphite filter (295 µm thick) and a Be window (254 µm thick) provide vacuum protection and attenuate only the low-energy photons. The beamline layout is shown in Fig. 1 ▶.

The double-crystal sagittally focusing monochromator [silicon (111)] is cryo-cooled with helium gas and provides a horizontal demagnification factor of 3.3:1. The last optical element before the experimental hutch is a meridionally bent fused silica mirror with two stripes (Pd coated and uncoated) vertically focusing the beam with a 6.6:1 demagnification ratio. A set of diagnostic tools are installed along the beamline. Downstream from the front-end apertures (FEAs) are two transmission-diamond white-beam position monitors developed in-house (Muller *et al.*, 2012 ▶), and a UHV QBPM foil (FMB Oxford, UK) (1.0 µm Cr) installed in 2009 is positioned immediately downstream of the monochromator. All beam-position monitors (BPMs) are left in the beam at all times and data from these devices are harvested to correlate with the users’ data collection and assess the stability of the beam during the experiment. The flux of the monochromatic beam is measured and optimized using ion chambers placed inside the hutch, one immediately after a Be window and the second in the path of the collimated beam on the diffractometer entrance arm.

**Figure 2:**
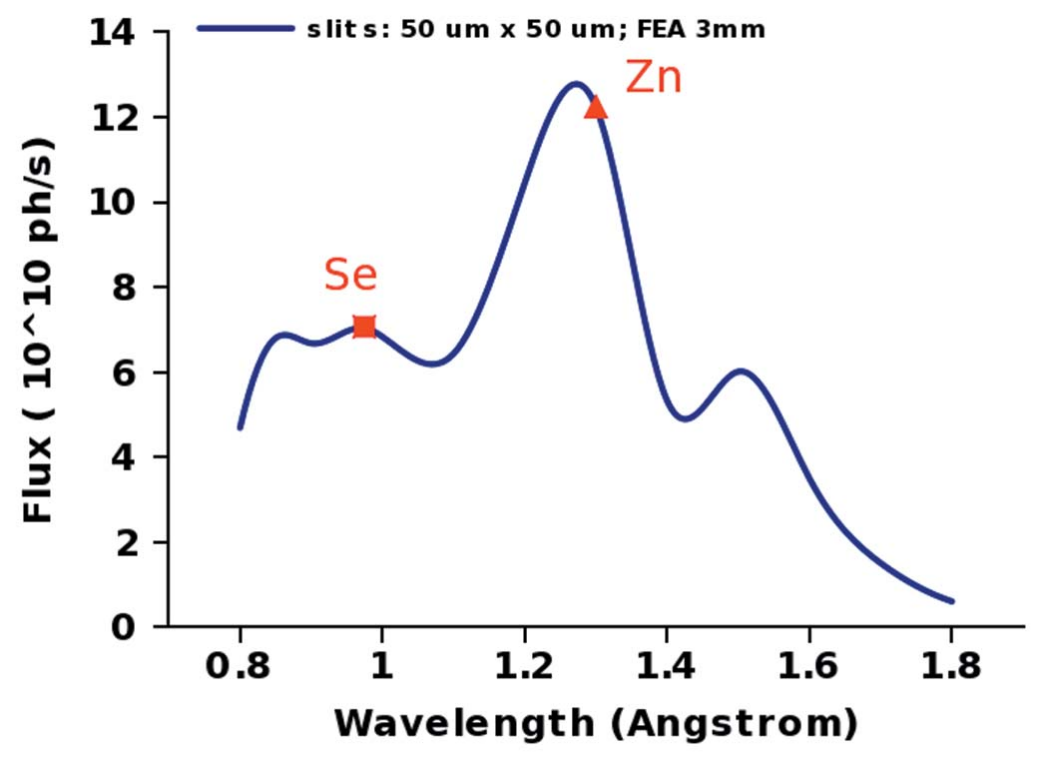
Flux at the beamline over the range of wavelengths available to the users. The slits in the endstation were set to 50 µm × 50 µm corresponding to the default setting for the beam size.

In the endstation, a micro-diffractometer (Crystal Logic, LA, USA) is installed on a separate stand from the detector table to avoid effects on the sample–beam alignment when changing the detector distance (Fig. 2 ▶). The air-bearing spindle has an independent vertical motion for accurate alignment with the beam in order to achieve reliable 20 µm × 20 µm mini-beam. We measured the ω rotation run-out of the spindle at the sample position with the standard mini-κ goniometer head to be better than 5 µm (peak-to-peak). The mini-κ simplifies hand mounting of the sample and provides rough re-orientation of the crystal axes in order to minimize overlaps when dealing with large unit-cell parameters (>250 Å) or high mosaicity (Brockhauser *et al.*, 2011 ▶). It enables 360° rotation around ω with a maximum offset of 42°. Its slim design prevents shadows on the detector and eliminates any collision restriction during data collection. In April 2011, the ADSC Q315r detector (Area Detector System Corporation, Poway, USA) was replaced with a pixel-array detector, the Pilatus 6M (Dectris, Baden, Switzerland), enabling shutterless data collection. The sample is kept at 100 K for standard operation (Oxford CryoSystems 700, Oxford, UK) and energy scans are performed using a Cyberstar YAP scintillator detector (FMB Oxford Ltd, Oxford, UK) to measure the X-ray fluorescence from the sample. The sample is visualized from a top view using a digital camera (Point Grey Research, CA, USA) resulting in a 270× zoom. The digital image is displayed on any browser window and the centering of the crystal is performed *via* a click-to-center procedure.

The PXRR fourth edition of a modified ALS-style automounter (Snell *et al.*, 2004 ▶) is installed at the beamline. Its original design using the 16-pin pucks is modified to also accommodate SSRL-style unipucks. A retractable cap dryer enables a smooth and fast transition over the 64 samples contained in a dewar. The drying time for each cap is set to 60 s, and this limits the duty cycle time for all 64 samples to 1 h.

**Figure 3:**
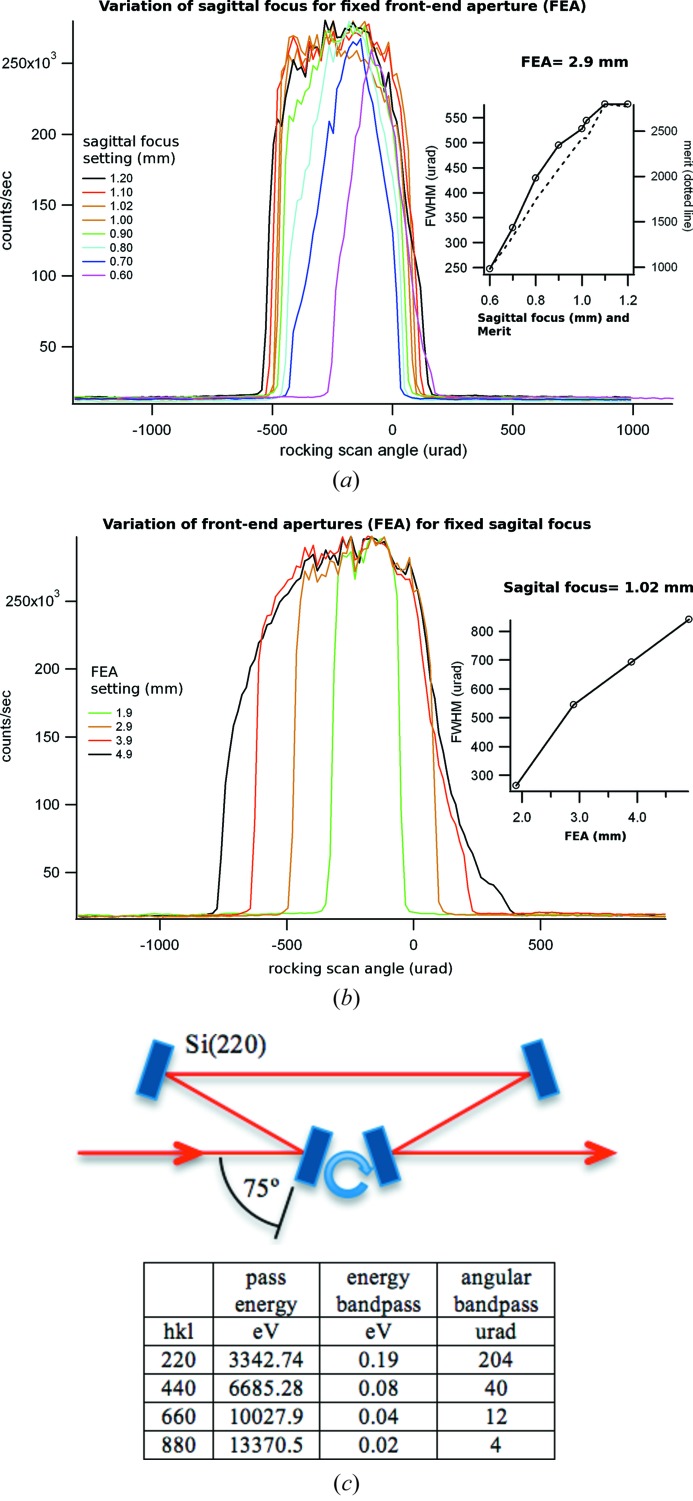
Two different methods of controlling the divergence of the beam. The measurements were performed at 10.057 keV with the four-bounce analyzer. (*a*) The front-end apertures were kept fixed at 2.9 mm and the sagittal focus on the second crystal was varied. The linear correlation of the merit (normalized counts for the ionization chamber) enables a quick assessment of the divergence. (*b*) Sagittal focus fixed at its optimal value for the energy used and the FEA were varied. (*c*) Diagram of the four-bounce analyzer apparatus.

The default native monochromatic beam in the hutch is set to 263 µm × 100 µm (FWHM, H × V) over a wide range of wavelengths (Table 1 ▶, Fig. 3 ▶) with a 0.3–0.5 mrad divergence. The beam is then slit down with variable apertures ranging from 2000 to 20 µm in either direction but typical experiments start with the preset values of 50 µm × 50 µm. Projects involving samples such as ribosome crystals benefit from a better collimated beam, so we made it straightforward to displace the focus further downstream towards the detector. In order to provide better and easier control of the divergence, extensive beam characterization measurements of the focused monochromatic beam were undertaken with a four-bounce double channel-cut crystal analyzer in the hutch (Dvorak *et al.*, 2009 ▶). This device consists of a pair of channel-cut Si(220) crystals arranged in a dispersive arrangement. The Bragg angle is designed at 75° (Fig. 3*c* ▶). As such, the analyzer will pass beam at approximately 3343 eV and all of the allowed higher harmonics with a very narrow energy and angular bandpass. The entire unit is then rotated with sub-microradian resolution by a custom-built rotational stage. This allows the divergence to be mapped out at one specific energy. Tuning the monochromator to several energies within its bandpass and repeating the analyzer scans allows the full energy/divergence characteristics of the beam to be mapped out. A photon beam energy spread of 9 eV (FWHM) was determined at 12 keV, using the sixth harmonic of the undulator source. The count rate from the ionization chamber in the hutch after the slits, divided by the ring current, is called the ‘merit’. This metric is displayed in the control software and is easy to monitor by users. The divergence can be customized either by adjusting the front-end apertures or by changing the curvature of the sagittally focusing crystal on the monochromator using preset values in the control software. Both options are easy to use and one only needs to monitor the merit in the hutch to monitor the divergence changes (Fig. 3 ▶).

## 3. Ancillary facility   

The computing power at the beamline is composed of two workstations to process the data while two more are dedicated for the control of the experiments. Most of the data processing software is available locally at the beamline. A laptop is present in the hutch for convenience to visualize the sample, but everything is controlled from the outside. The data storage is 20 TB over a 2 Gbit connection with a four-node cluster enabling processing Pilatus data at its fastest rate (12 Hz). For offline computing, X25 also shares with other MX beamlines three cyber café spaces where a dozen workstations with dual head monitors are available at any time. Data from any of our beamlines are available from these computers. These quiet areas are used during visits by individuals in the group not required at the beamline while collecting. An extensive set of crystallography software is available at these computers but these areas are mainly used to finish work and back up data after allocated beam time has ended.

A liquid-handling device is available at the NSLS for PXRR users trying to figure out new crystallization conditions. The Echo 550 (Labcyte, Sunnyvale, USA) is available to not only set up crystallization trays using very small volumes of protein (∼50 µL initial volume for 2000 conditions) but it can also be used to mount crystals onto meshes to bring to the beamline. Very small crystals of a few micrometers or less benefit from reduced background from 2.5 nL of mother liquor that is transferred with each crystal. Large crystals also benefit from the speed and automation of acoustic crystal mounting. Crystals of all sizes can be combined with chemicals from a fragment library, either *in situ* (by combining the crystals and chemicals directly on the data collection mesh) or in crystallization wells.

### 3.1. Control, data acquisition software and database   

We run EPICS on RTEMS on a VME crate for our motor control and counters, and Linux-based IOCs for almost everything else. Our goal is to put as much as possible under EPICS in order to minimize code and maximize flexibility. Since 2005, our entire system has been integrated with a PostgreSQL LIMS system, called PXdb. This is the portal for users to request beam time, manage their projects, and describe samples in their automounter pucks or canes. Most importantly, they can perform ‘sweep queries’ to look at any of their data runs collected since 2005. These have detailed logs that include jpegs of their diffraction images, crystal pictures, beam intensity plots, BPM plots, and other information. Their diffraction images can be downloaded to their home institution *via* these queries as soon as their sweep completes. The data are available until erased from our disks (two to four weeks) but the log is kept indefinitely. This is particularly popular with Mail-in and Qmx users (see §5.2) since they can start working on their data as soon as collected. Despite having to control slightly different hardware from one PXRR beamline to the other, the GUI remains the same throughout, making it easy for users to migrate from one beamline to another. This data collection software which users interact with is a Python-based system called *CBASS* (Skinner *et al.*, 2006 ▶). This software is very adaptable due to its simplicity with only 13K lines of code spread over 20 files. Great efforts are put in place to encourage users to take advantage of the options implemented at the beamline in order to maximize the use of the beamline and beam time.

## 4. Data collection   

**Figure 4:**
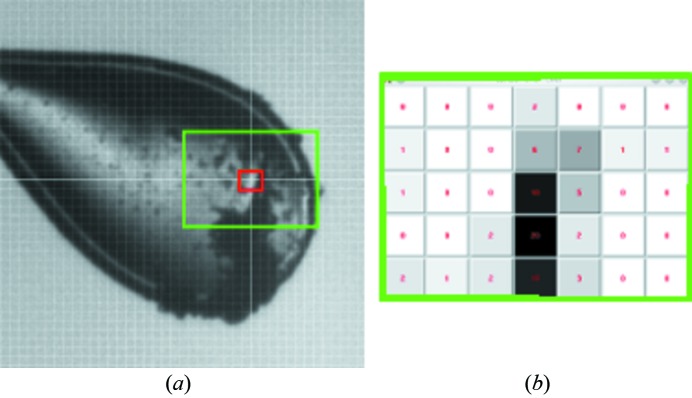
(*a*) Typical view of a loop where glare and ice obscure the sample location. The red box is the beam size used, set to 30 µm^2^, while the green display is the area rastered. These parameters can be customized by the users. (*b*) Pop-up map with grayscaled squares corresponding to each position of the raster scan which are saved in the PXdb for further reference. Clicking on the desired square will reposition the area in the beam and the corresponding diffraction image will pop up to be examined. An offset can be defined *via* the PXdb web interface to remount and reposition the sample to the desired location at a different time.

One of the most common fears is to misalign the crystal and the X-ray beam while collecting. A quick way to visually verify the alignment of the beam-spindle is to mount a large loop with a minimum thickness of a 50% glycerol:100 m*M* cacodylic acid solution. The size and shape of the beam become visible as a dark mark after a few seconds exposure time. The regular visual alignment of samples is often impaired by its surroundings and the lighting set-up. With the advent of automounters at the MX beamlines and a push for automated sample alignment, it is necessary to go beyond simple loop centering for finding the small crystals. We developed and implemented an alternative way to locate the crystal by performing an X-ray grid scan across a specific area of the sample similar to what other facilities do (de Sanctis *et al.*, 2012 ▶; Hilgart *et al.*, 2011 ▶). First the flat face of the loop is orientated perpendicular to the beam using *XREC* (Pothineni *et al.*, 2006 ▶). A very short exposure time (0.1 s) is recommended; crude diffraction data are harvested only in the central region of the detector where low-resolution data would appear. This minimizes radiation exposure and avoids potential ice rings in the diffraction pattern. The software uses the program ‘module_peaksearch’ written by C. Nielson of ADSC to count the peaks >5σ from each position of the grid. The analysis is carried out as the raster scan is performed and results in an interactive grayscale map to find the crystal (Fig. 4 ▶). This operation is repeated 90° away. Once positioned on the crystal, standard exposures are taken and estimated resolution is calculated and results are automatically written to the sweep log (PXdb). Other crystallography software such as *DISTL* (Sauter *et al.*, 2004 ▶) or *EDNA* (Leslie *et al.*, 2002 ▶) were initially used to provide detailed crystallographic information about the diffraction. Since most of the grid does not yield diffraction, we opted for a faster preliminary analysis. The total time to perform this operation with the default parameters is less than 1 min.

The default set-up at the beamline is to have the beam slit down to 50 µm × 50 µm but adjustment to larger or smaller beam is encouraged. Having the aperture size (beam size) displayed as a red box on the centering display entices users to try to tailor the beam to their crystal size. For long thin needles, a vector scan (a.k.a. a helical scan) is available as a data collection mode. The users only need to define the starting point and the end point of the crystal and specify the number of frames per step.

Since a 360° data collection is routinely performed in less than 10 min, keeping track of the data and organizing it becomes a major part of the experiment. An easy fix to avoid struggling with beam center coordinates is addressed by taking an automatic direct beam for every data collection (a sweep with >20 frames) and adding this frame to the data collection directory. The frame is used to ascertain that the coordinate values used match those of their processing software of choice. This direct beam shot also enables us to write the correct beam center coordinates [in pixel values compatible with the *XDS* software (Kabsch, 2010 ▶)] in the header of each frame. An automatic data reduction procedure, using the *xia2* software package (Winter, 2010 ▶), is in place and triggered on demand.

A major challenge is to be able to collect enough data before radiation damage occurs. The use of *RADDOSE* (Paithankar *et al.*, 2009 ▶) is crudely implemented with the default size of the crystal being the aperture size. The lysozyme cell parameters are used as default and the dose corresponding to the chosen data collection parameters and the number of frames at which the Henderson limit would be reached is displayed before the sweep is started. For more accurate dose values, users can input values corresponding to their sample.

## 5. Facility access   

Beam time at the NSLS can be obtained by several different mechanisms all running in parallel. These will mostly vary in the lead time before being scheduled, ranging from three months to a few days.

Users must complete typical safety and standard cyber security training to gain access to the facility. In the case of remote access an RSA securID needs to be obtained from BNL to gain remote computer access (http://www.bnl.gov/cybersecurity/rsa/).

### 5.1. General user time   

Several access modes are being used simultaneously to obtain beam time at any of the PXRR beamlines. The general user (GU) program from the NSLS using the PASS system (Proposal Allocation Safety Schedule) requires a formal project description to be reviewed by an external committee and results in 24 h blocks allocated for the next trimester. This proposal is active for two years. X25 has 50% of the available beam time reserved for GU. RapidAccess works with an existing active proposal for the current semester if GU time is still available. Proprietary users are encouraged to use either mode.

### 5.2. PRT/PXRR access   

The PXRR also allocates the remainder of the beam time *via* the PXdb. Users login as their group account, fill out project information and the scientific beamline staff will review their request for feasibility. A request for RapidAccess, Mail-in or Qmx beam time is then evaluated. The two first modes allocate a 16 h block of time (overnight) or shorter 2–4 h blocks during daytime. This can be for on-site or remote visits.

The Mail-in program (Robinson *et al.*, 2006 ▶) started a decade ago and has remained a very popular access mode since then. Scientific staff receive samples and perform the desired or necessary experiments in a collaborative mode with the users.

We are anticipating the asynchronous remote access mode, Qmx, to meet the need of users of the NSLS-II facility. Samples are sent to be systematically screened. Even though the local staff load the puck in the automounter and define the queue, the screening and data collection are fully software automated. The beamline and data collection defaults are initially set to optimize robustness in the procedures rather than speed; for example, the raster scan for finding the crystal is larger than the regular one. If triggers are defined such as resolution limit, data collection can proceed immediately after the screening if the trigger value is met. Otherwise Qmx users can evaluate the results the next day or when convenient, and define subsequent experiments to be performed for each sample; the pucks will then be queued for the next available time on the beamline. All results and interactions (re-screening at a different location in the loop, collecting at a specific wavelength, selecting specific wedges) are performed *via* PXdb. For any of these access modes, time can be obtained within a week or two as we promote shorter but more frequent access time to the facility.

## 6. Productivity   

**Figure 5:**
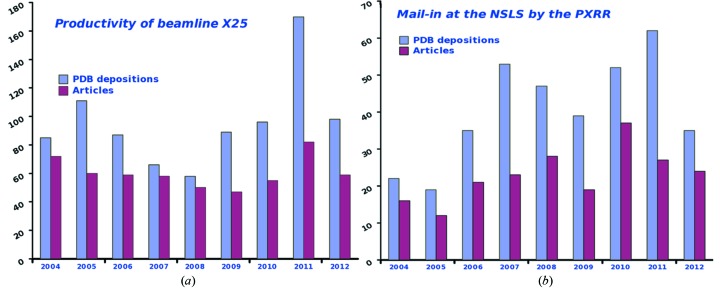
(*a*) PDB depositions and articles from beamline X25 over the years since it was dedicated to the MX mission. Data were taken from the Biosync website (http://biosync.sbkb.org/) and the NSLS publications list (https://pass.nsls.bnl.gov/publications/search.asp). The dip in 2008 corresponds to the upgrade period. (*b*) Number of articles and PDB deposition resulting from Mail-in work carried out at all PXRR beamlines. For a list of these publications, visit http://wwwpx.nsls.bnl.gov/Mailin.html.

X25 has been productive in publications and Protein Data Bank (PDB) depositions (Fig. 5 ▶) over its existence and accommodates around 100 different groups a year with roughly 11000 crystals mounted per year. Synergy between beamlines and beam time allocation is in place to better serve the user community. Screening can occur at one beamline and data collection at another. This is reflected in the publications list where 60% of the PDB depositions indicated that work was performed on at least two beamlines at the NSLS (https://pass.nsls.bnl.gov/publications/search.asp). Coordination amongst MX beamlines and complementary experiments such as correlated spectroscopy at X26C (Orville *et al.*, 2011 ▶; Stoner-Ma *et al.*, 2011 ▶) or SAXS at X9 (Allaire & Yang, 2011 ▶) is in place to better schedule beam time for users.

## 7. Highlights   

### 7.1. Acoustically mounted microcrystals yield high-resolution X-ray structures (Soares *et al.*, 2011 ▶)   

Time and effort are allocated at X25 to the scientific staff to develop projects relevant to future methods for collecting data of micrometer-sized biomolecular samples. One innovative method uses acoustic droplet ejection to enable handling a very small volume (nL) of highly concentrated micrometer-sized crystals. A large quantity of these crystals is required to obtain a full dataset due to limited data obtained from each crystal. This work demonstrated that structural information can be obtained from a slurry of insulin or lysozyme crystals (5, 10 or 20 µm size) which would have been discarded if traditional manual mounting methods had been used. The crystals were acoustically ejected on a mesh from a slurry. These meshes contained several crystals that were found using the 20 µm × 20 µm mini-beam and a raster scan, and then wedges were collected. This work helped develop beamline features such as the raster scans, data collection set-ups and database implementations to keep up with the experiments. It brings us a step closer to the implementation of novel methods for sample delivery to the beam that are needed for the newer storage rings being built all around the world. (Data collected at beamlines X12B, X12C, X25, X29A and at the APS 23-ID-D.)

### 7.2. Extensive mutagenesis of the HSV-1 gB ectodomain reveals remarkable stability of its postfusion form (Vitu *et al.*, 2013 ▶) (PDB 4sh1, 3nwa)   

The mechanism of herpesvirus cell entry requires numerous interactions between the host-cell receptor and the viral protein machinery. The entry mechanism is complex and fusogenic functions are carried out by conformational changes of multiple viral proteins including the glycoprotein B (gB). The structure of the postfusion state of gB is well characterized but the conformation of the prefusion state of gB remains unknown. Efforts are ongoing to identify mutation to destabilize the gB which could adopt the metastable prefusion conformation. Engineered point mutations at several key residues were performed and tested by cryoEM and other methods. The structures of these mutants were characterized at beamline X25. Quite often these mutations generated only small crystals having a large unit cell (*P*3, *a* = *b* = 117 Å, *c* = 320 Å) which diffract to 3.1 Å resolution. So far more mutants are still needed and required to be characterized at X25 for the identification of the prefusion conformation of gB. Understanding the conformational change will be critical in the development of new drugs for antiviral therapy. This work is a good exemple of the tight collaboration with the Mail-in program to help with the fast turnaround of a project.

## 8. Discussion and conclusions   

Our very flexible access mode and streamline data collection software allows our set of beamlines to be competitive and as productive as other beamlines at more modern synchrotrons in the world. The great majority of users are local and come from a region encompassing New York City to Boston. The fast turnaround for scheduling, sometimes within hours of a request, accelerates the progress of their research projects. The presence of px-operators during off-hours contributes to a high quality of support for the users. Having a great synergy with X29, which can accommodate the load imposed by user access, enables X25 to pursue development projects such as new sample delivery methods (Roessler *et al.*, 2013 ▶) in order to be ready to implement at the MX beamlines at the future NSLS-II facility.

## Figures and Tables

**Table 1:** X25 beamline characteristics

Source type	Mini-gap undulator, 55 × 18 mm periods
Mirror; coating stripes	Vertical focusing; Pd, uncoated
Monochromator	Cryo-cooled double-crystal sagittally focusing Si(111)
Energy range used (keV)	13.4–6.8
Wavelength range used (Å)	0.92–1.8
Focused beam size (native, default setting) (V × H) (µm)	100 × 263, 50 × 50
Flux (no slits, slits with default settings) (photons s^−1^ at 1.1 Å)	9.8 × 10^11^, 6.7 × 10^10^
Typical monochromatic beam divergence (mrad)	0.3–0.5
Goniometer	Mini-κ
Cryo capability	Yes
Sample mounting	Manual/ALS style robot/ADE
Detector type, model	Pixel array, Pilatus 6M
2θ capabilities	None
